# A 35-year-old father with persistent Mullerian duct syndrome and seminoma of the right undescended testis: a rare case report

**DOI:** 10.1186/s40792-021-01354-w

**Published:** 2021-12-27

**Authors:** Marah Mansour, Abdullah Fattal, Yassamine Ouerdane, Tamim Alsuliman, Omar Kanjawi

**Affiliations:** 1 Faculty of Medicine, Tartous University, Tartous, Syrian Arab Republic; 2 Faculty of Medicine, Aleppo University, Aleppo, Syrian Arab Republic; 3 Faculty of Medicine, SaadDahlab University, Blida, Algeria; 4 Hematology and Cell Therapy Department, Saint-Antoine Hospital, AP-HP, Sorbonne University, Paris, France; 5 Department of General Surgery, Tishreen Hospital, Manbij, Aleppo, Syrian Arab Republic

**Keywords:** Persistent Müllerian duct syndrome, Seminoma, Exploratory laparotomy, Cryptorchidism

## Abstract

**Background:**

A persistent Müllerian duct syndrome is a rare disorder of sexual differentiation characterized by the presence of the female reproductive system in a normal male.

**Case presentation:**

Herein, we report a case of a 35-year-old father with persistent Müllerian duct syndrome and seminoma in the right undescended testis. The exploratory laparotomy was performed and revealed a mass in the right undescended testis and Müllerian duct structures.

**Conclusions:**

For patients with cryptorchidism and inguinal hernia, the persistent Müllerian duct syndrome should be considered, and radiological evaluation of the genitourinary system is recommended for early diagnosis of persistent Müllerian duct syndrome. The persistent Müllerian duct syndrome is usually detected during a surgical operation, and it is considered a risk factor for developing testicular malignancies.

## Background

Persistent Müllerian duct syndrome (PMDS) is a sexual development disorder characterized by the presence of female reproductive organs in individuals with both normal chromosomes (46, XY) and a normal phenotype of a male. It could be accidentally found during orchidopexy, laparotomy, or routine inguinal hernia repair in patients presenting with cryptorchidism (undescended testis).

Usually, one testis is typically positioned, and the other is undescended. However, it is conceivable to be bilateral as well [1, 2]. The persistence of Müllerian ducts (MD) in these genetically normal males may be explained by the incapacity to produce the anti-Müllerian hormone (AMH) by Sertoli cells or a defect in AMH type II receptor (AMHR-II), since this hormone causes MD regression [3]. Cryptorchidism is a risk factor for developing testicular cancers, and it was reported to be associated with PMDs [4, 5]. So far, few cases of PMDs associated with seminoma have been reported.

## Case presentation

**Fig.1:**
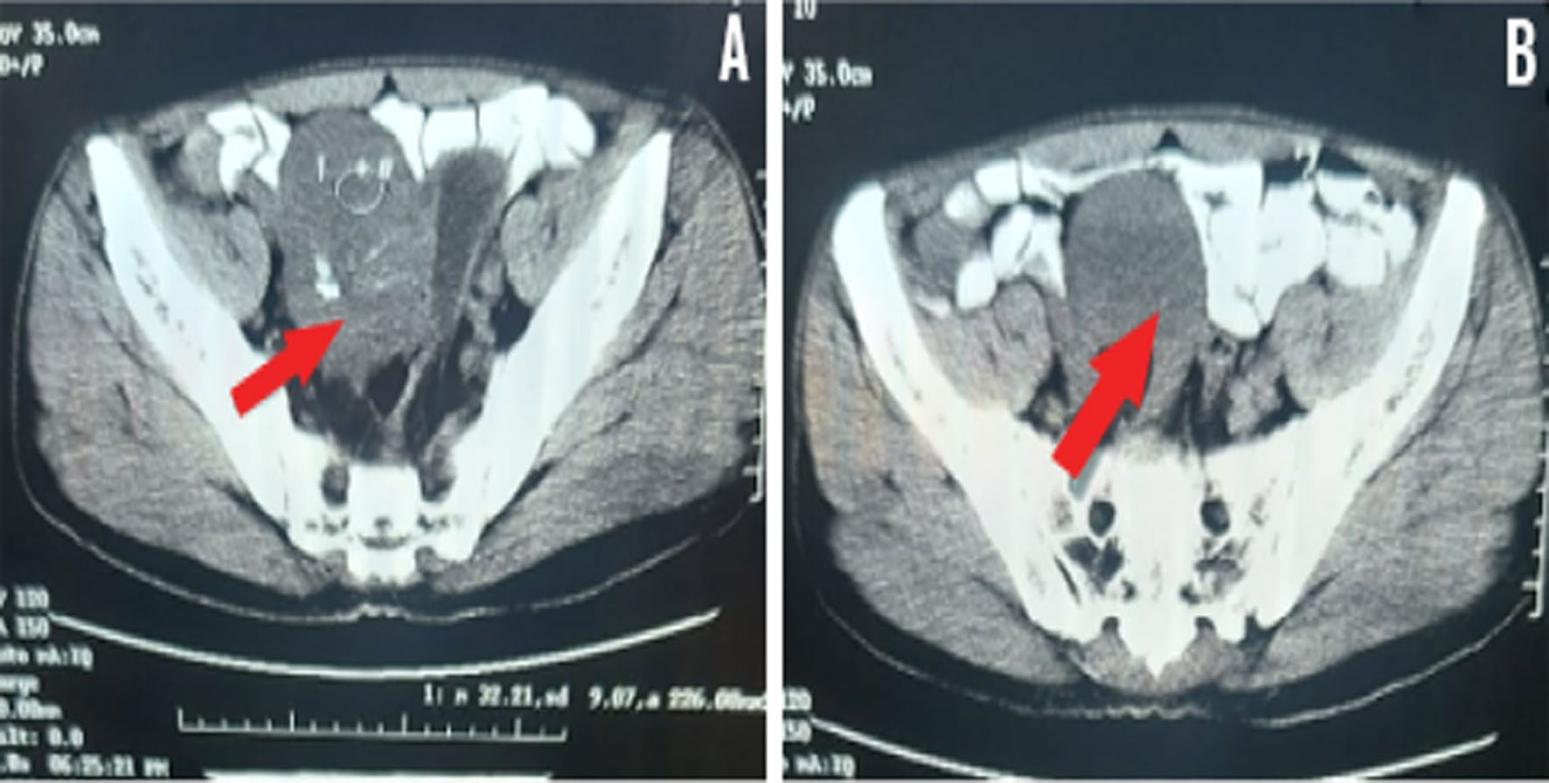
**A**, **B** Axial pelvis CT scan with intravenous injection of contrast material shows the pelvic cyst (red arrow)

A 35-year-old mute father with one child presented to the Department of General Surgery with persistent spastic abdominal pain in the right iliac fossa (RIF) treated with antispasmodic and analgesic medications. The patient has a typical male appearance with regular secondary sexual characteristics and a medical history of right inguinal hernia repair 15 years ago. Apart from the abovementioned medical history, an ultrasound (US) demonstrated a large echogenic mass located in the RIF. No clinical signs of acute appendicitis were found which excluded the diagnosis of appendicitis. Consequently, a computed tomography (CT) scan was performed and revealed a hypodense homogeneous mass between the cecum and the urinary bladder with a diameter of 8 cm compressing the latter and allowing the diffusion of the contrast product (Fig. 1).

**Fig.2:**
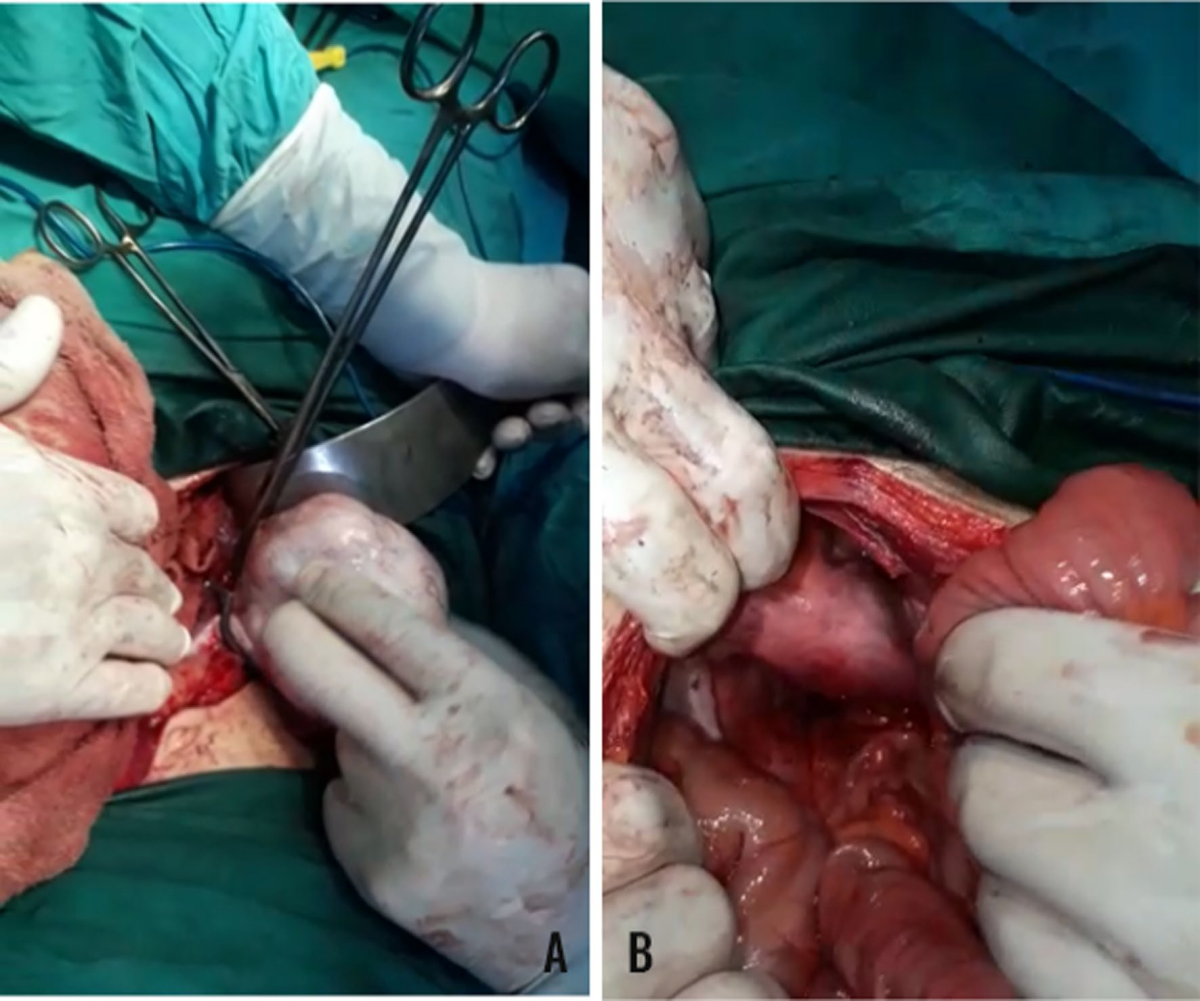
**A** Pelvic cyst mass in the Iliac fossa and **B** uterus found during the laparotomy procedure

A large internal iliac lymph node exceeding 1 cm of diameter was also observed. Furthermore, evaluating the right upper hypochondriac region with a CT scan demonstrated a gallbladder containing a small stone. A laparotomy procedure was performed. Exploring the abdominal iliac fossa revealed a dermoid cyst in an undescended right-sided testis, resulting in a perioperative diagnosis of an undescended testis tumor. Moreover, during laparotomy, a globular structure resembling the uterus with the cervix, Fallopian tubes, and vagina were found (Fig. 2).

**Fig.3:**
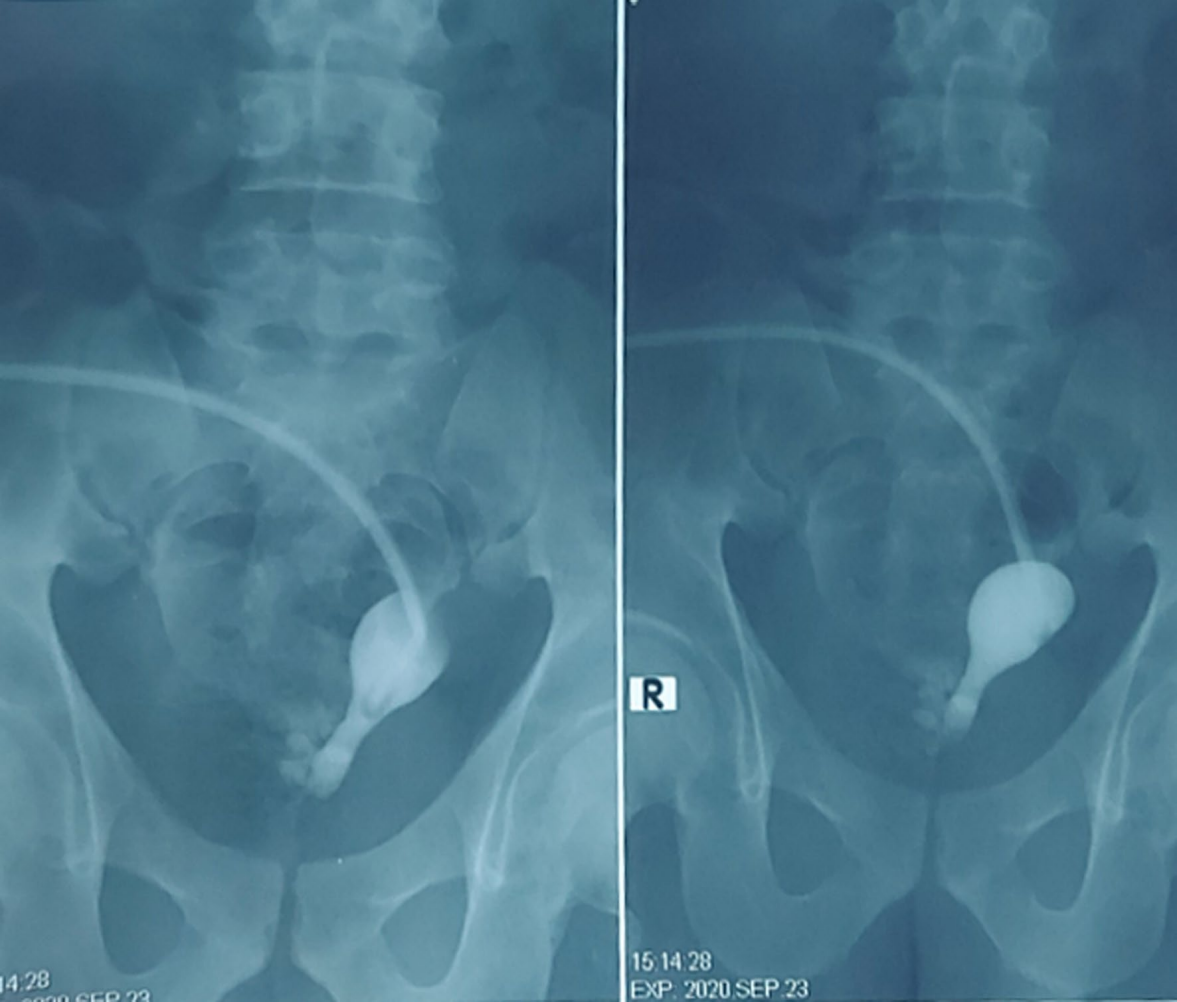
Uteroscopy image shows uterus and right infundibulum attached to the visceral abdominal wall

However, ovaries were not found. A uterine catheter with a radio-contrast agent to explore the uterus depth was introduced, and a right infundibulum was discovered attached to the visceral abdominal wall (Fig. 3). An orchiectomy for the right undescended testis was performed, as well as resection of the associated mass. Lymphadenectomy was not performed due to the proximity of the node to the iliac artery. Ten days later after obtaining the written patient content. The second surgery was performed, total excision of the uterus with the Fallopian tube, cervix, infundibulum, and cholecystectomy, were conducted.

**Fig.4:**
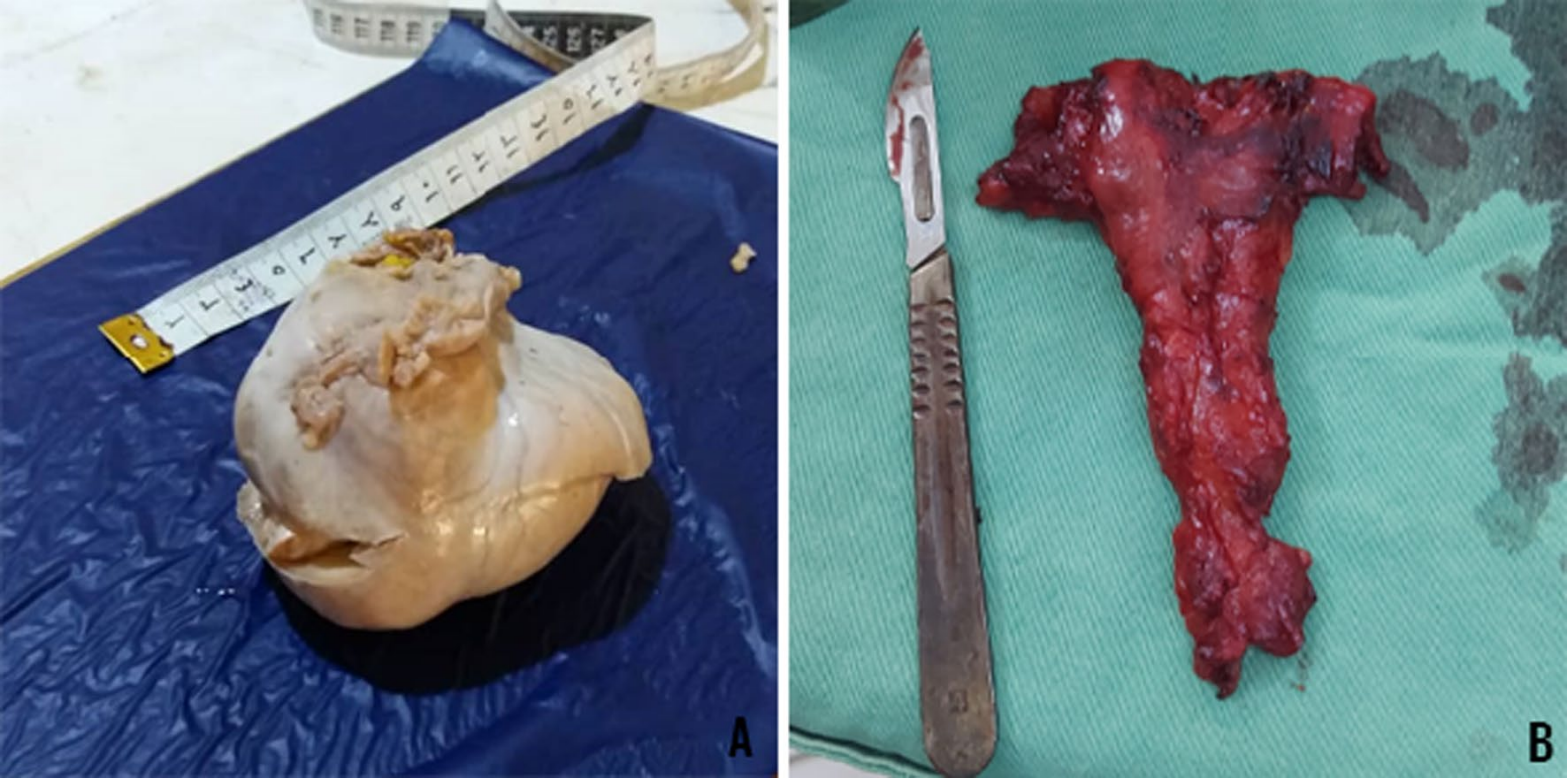
**A** Mass of the well-circumscribed testis, with smooth gray mass, and attached tube-like structure. **B** Rudimentary uterus

During surgery, a mucous liquid in the uterus cavity, not exceeding 50 ml, and a swollen pouch of Douglas were found. The specimen showed a mass of well-circumscribed testis, with smooth gray mass, and an attached tube-like structure. Weighs 239 gm measured ~ 10 × 75 cm (Fig. 4). Cut sections revealed a tan/white friable surface. Cut sections of the mass showed sheets and lobular configuration of germ cells with hyperchromatic nuclei and prominent nucleoli surrounded by thin fibrous septa infiltrated by lymphocytes. No hemorrhage or necrosis was noticed. The nearby regions showed hyalinized seminiferous tubules.

**Fig.5:**
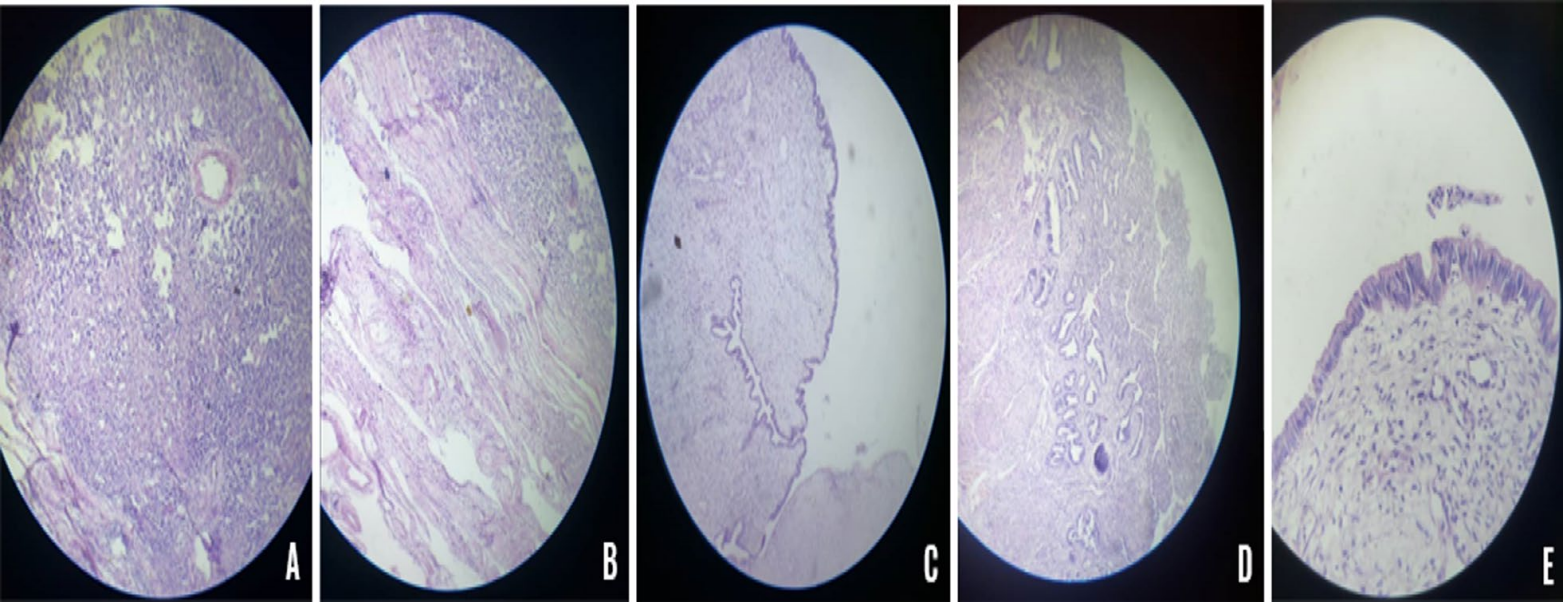
**A** Sheets and lobular configuration of germ cells with hyperchromatic nuclei and prominent nucleoli surrounded by thin fibrous septa infiltrated by lymphocytes. **B** Hyalinized seminiferous tubules. **C** Fallopian tube tissue surrounded by fibro-fatty tissue contains rete testis and efferent ductules. **D** Atrophic endometrium and myometrium. **E** Cervix with ulcerated endocervical mucosa, and seminal vesicles tissue

The histopathological study confirmed the features of a classical seminoma stage T1b. The tunica albuginea and the epididymis were free (Fig. 5). Throughout second surgery, the specimen of the pelvic mass-like uterus shape revealed a rudimentary uterus with a cervix that weighs 60 gm and measures ~ 11 × 5 × 3 cm, the body ~ 5 × 5 × 3 cm, and the cervix ~ 6 × 2 cm (Fig. 4). Cut sections revealed an endometrial-like surface. The histopathological study demonstrated atrophic endometrium and myometrium of the uterus. In the cervix, an ulcerated endocervical mucosa was observed.

Furthermore, seminal vesicle tissue is noticeable on the wall. On one side of the uterus, the Fallopian tube tissue surrounded by fibrous-fatty tissues, including rete testis and efferent ductules were detectable. The histopathological examination overall confirmed the presence of both seminoma on the right side undescended testis and uterus, cervix, Fallopian tube (persistent Müllerian duct structures) (Fig. 5). The final diagnosis was a seminoma of the undescended testis with PMDS. Post-operation, the patient's condition remained stable with no evidence of recurrence. He was assigned to the cancer center and he responds well to chemotherapy, his clinical conditions are stable the patient is seen twice yearly.

## Conclusions and discussion

PMDS was first described in a male with an inguinal hernia in 1939 by Nilson, presenting as hernia uteri inguinal. It may be caused by an absence of AMH released from Sertoli cells of the male fetus from the seventh week of gestation, it is responsible for the regression of the Müllerian duct. When AMH is not secreted, that may lead to the persistence of female reproductive organs in the males with PMDS [6, 7].

Two anatomic variants have been described: male and female. The male type is most common, and it is classified into two subcategories: hernia uteri inguinal and transverse testicular ectopia. The majority of cases present with unilateral cryptorchidism, and contralateral inguinal hernia [8]. In this case, we demonstrate a rare type of pseudo-hermaphroditism in males characterized by extra female genital organs present in a normal 46XY genotypical and phenotypical father. These organs represent MD structures (the uterus, Fallopian tubes, and upper two-thirds of the vagina). However, radiological findings only revealed the right Fallopian tube in this syndrome (Fig. 3) [9].

PMDS patients are rarely fertile and most of them usually suffer from infertility and inguinal hernia. Causes of infertility are various as testis hypoplasia, and ejaculatory duct obstruction due to compression by MD structures. In comparison, our patient was married and had a child [10].

The diagnosis of PMDS is often controversial, because there are no specific clinical symptoms, it is discovered incidentally either hernia repair or surgery for undescended testes. The risk of malignant transformation increases after puberty; therefore, early diagnosis should be recommended (Fig. 5) [11, 12].

Seminoma is the most common testicular germ-cell tumor. In PMDS, the incidence of malignant transformation in the undescended testis is similar to the incidence of testicular carcinoma in patients without PMDS. Moreover, Müllerian malignancies are more probable to develop than testicular cancer in PMDS [12, 13].

Usually, the undescended testis is detected in the inguinal channel or intra-abdominally, as in our case the right undescended testis was situated intra-abdominally and the left one was in the scrotum. When testicular malignant is suspected the appropriate surgical procedures of orchiectomy followed by removal of MD structures may be discussed [14].

As a long-term follow-up, the palpation of the testis and radiological examinations should be mandatory for any inconsistencies in patients with PMDS [12]. In stage T1b seminoma a single dose of the carboplatin area under the curve 7 (AUC7) was chosen to be the standard treatment for stage I seminoma rather than the adjuvant radiotherapy according to the results from MRC TE19/EORTC 30982 Study [15]. In our case, the patient consent was requested to perform the excision of the Mullerian remnants which were first being discovered during the laparoscopic procedure. This is considered a fundamental step due to the inconclusive guidelines of performing a surgical resection of the mass or just long-term monitoring [11, 12].

Of note the resection of the Mullerian remnants is not standardized as a treatment approach due to the high risk of malignancy; however, a recent outcome analysis showed the effectiveness of performing a laparoscopic approach to treat the patients with PMDS [16].

This publication aims to emphasize the importance of profound clinical examination that may help the diagnosis of PMDS associated with seminoma, even in males with typical appearance and secondary sexual characteristics. The correlation between these two diseases is still unclear. Further diagnostic procedures are required while dealing with an inguinal hernia and cryptorchidism patients to early detect PMDS and to prevent relevant complications.

## Data Availability

Not applicable. All data (of the patient) generated during this study are included in this published article and its supplementary information files.

## Abbreviations

PMDS: Persistent Müllerian duct syndrome
MD: Müllerian ducts
AMH: Anti-Müllerian hormone
RIF: Right iliac fossa
US: Ultrasound
CT: Computed tomography
(AUC7): Area under the curve 7

## Declarations

### Guarantor

Omar Kanjawi is the guarantor of this work.
